# Consumption

**Published:** 1880-07

**Authors:** 


### HALL'S JOURNAL OF HEALTH.

OUR LEGITIMATE SCOPE IS ALMOST BOUNDLESS: FOR WHATEVER BEGETS PLEASURABLE AND HARMLESS FEELINGS, PROMOTES HEALTH; AND WHATEVER INDUCES DISAGREEABLE SENSATIONS, ENGENDERS DISEASE.

**We aim to show how disease may be avoided, and that it is best, when sickness comes, to take no medicine without consulting an educated physician.**

### CONSUMPTION

Is a disease which destroys one out of every six grown persons, which invades every family in the civilized world, which carries to the grave, from New York city alone, in the space of a single year, more than three thousand persons. Such a disease ought to be understood, at least as to its general features. Every intelligent individual ought to have a general idea of its nature, its causes, and its prevention. Such a result will be of slow attainment. The thoughtful few will give it attention, which will gradually force itself on the masses, at least so far as the general principles of prevention are concerned.

We believe that, within a few years, there has been a progressive popular intelligence on the subject, and that more correct views prevail in reference to its nature, than at any previous time. Now and then a man of extraordinary intelligence and practical good sense makes his appearance, such as Moreton, of Philadelphia, Wooster, of Cincinnati, and Norcom, of Carolina; but they have passed away, and in a whole lifetime succeeded in impressing their views upon a chance individual here and there.

Two sentiments pervaded the theory and practice of these excellent men—and they were all contemporaries, but most probably wholly unknown to each other. Moretcn was widely known; we never saw the amiable Wooster's name in any printed book; and we ourselves never heard of Norcom until years after his death. They believed that—

1st. No medicine known to them had any uniform appreciable good effect in common consumption of the lungs.

2d. Without a free exposure to out-door air, no case of consumption ever was cured.

These same sentiments are now taking hold of the medical minds of Great Britain, with several remarkable additions: that—

1st. Sea voyages hasten the fatal result of consumptive disease.

2d. Seeking a milder climate precipitates a like result.

3d. The maintenance of an equal artificial temperature only aggravates the disease.

"There is an antecedent state of disordered health, which, as a causative agent, originates that altered state of the blood which produces tubercle. To this part of our subject, I would entreat your earnest attention. It is not only the key to the disease, but it is the hopeful period for treatment—the critical time in which we may check the inroads of the most fatal of all affections incident to the human frame. Feel no hesitation in saying that the earliest symptom of consumption is 'WASTING;' it precedes cough, and spitting of blood, and hectic, and all the physical signs; * * * hence the second symptom is *debility*."

The diseases to which the lungs and their proper appendages are liable, are Asthma, Bronchitis, Consumption, Laryngitis or Throat ail, Croup, Pleurisy, Inflammation of the Lungs, Congestion of the Lungs, Quinsy, &c. All these diseases arise from two causes:

1. Changes of temperature.

2. The failure to keep the breathing apparatus in vigorous, full, healthful operation, by a sufficient amount daily of exertive exercise in the open air. By a wise attention to this second cause of lung diseases, the former will cease to be a cause, except in occasional cases. Not only so, threatened consumption may be effectually warded off, in the vast majority of cases, by the proper adaptation of daily out-door exertive exercise to the requirements of the system, as indicated by the condition of the pulse, the heart, the breathing organs, of which the physician ought to be the standing judge. For, when a man is an invalid, the amount of food, air, and exercise which he needs, requires as much of medical intelligence, experience, and skill, as would the judicious exhibition of medicine.

*Deficiency in breath, and strength, and flesh,* are the first far-off symptoms of consumption, coming on as they do long before cough, and spitting of blood, and pains about the chest. We believe that, in the vast majority of cases, the foundation—the first symptoms of consumption in both sexes, is laid at about the age of eighteen or twenty years, and that, if medical attention were secured at this age to children, by which a critical examination could be made as to those points, and corresponding action follow, the mortality from this disease would be largely diminished at once. To neglect these earliest symptoms of consumption, is like neglecting looseness of bowels in Asiatic cholera, which is nothing short of almost certain death.

No one seems to have the slightest fear of consumption now-a-days, unless there is some cough, when the fact is, some persons have observed no cough at all until within three weeks of death, and on examination the lungs have been found a mass of rottenness. In the vast mass of cases, cough is a telegraphic signal that the train has been fired, that tubercles have been deposited, and are taking on irritative and inflammatory action, which is the stage immediately preceding actual decay of the lungs; whereas, if short breath, wasting of flesh, and general weakness, were considered, as they really are, the *avant couriers* of the disease, and corresponding action had, three fourths of those who now die of consumption could ward off the disease indefinitely. Hard-earned thousands, yes millions, often go to distant relations, as heartless as they are greedy, which else would have fallen to natural heirs, if, instead of nursing children from seventeen to twenty years, the moment they seem to become "*weakly*," by consigning them to feather beds, and furnaced rooms, and closed carriages, afraid as death of a single puff of fresh out-door air, lest it should give them a cold—we say a far different result would have followed the hard mattress, the cold sleeping-room, the frequent walk and daily horseback ride, early and late, of from five to thirty miles a day, in spite of all obstacles of wind, and storm, and dust, and snow, and hail—of zero or of broiling heat.

